# Complete loss of E-cadherin expression in a rare case of metastatic malignant meningioma: a case report

**DOI:** 10.1186/s12883-023-03450-w

**Published:** 2023-11-04

**Authors:** Dominik Lisowski, Philipp E. Hartrampf, Natalie Hasenauer, Vera Nickl, Camelia-Maria Monoranu, Jörg Tamihardja

**Affiliations:** 1 Department of Radiation Oncology, University Hospital Würzburg, Josef-Schneider-Str. 11, 97080 Würzburg, Germany, Germany; 2 Department of Nuclear Medicine, University Hospital Würzburg, Würzburg, Germany; 3 Department of Neurosurgery, University Hospital Würzburg, Würzburg, Germany; 4 Department of Neuropathology, Institute of Pathology, University of Würzburg, Würzburg, Germany

**Keywords:** Beta-catenin, E-cadherin, Meningioma, Peptide receptor radionuclide therapy (PRRT), Radiotherapy

## Abstract

**Background:**

Hematogenous tumor spread of malignant meningiomas occurs very rarely but is associated with very poor prognosis.

**Case presentation:**

We report an unusual case of a patient with a malignant meningioma who developed multiple metastases in bones, lungs and liver after initial complete resection of the primary tumor. After partial hepatic resection, specimens were histologically analyzed, and a complete loss of E-cadherin adhesion molecules was found. No oncogenic target mutations were found. The patient received a combination of conventional radiotherapy and peptide receptor radionuclide therapy (PRRT). Due to aggressive tumor behavior and rapid spread of metastases, the patient deceased after initiation of treatment.

**Conclusions:**

E-cadherin downregulation is associated with a higher probability of tumor invasion and distant metastasis formation in malignant meningioma. Up to now, the efficacy of systemic therapy, including PRRT, is very limited in malignant meningioma patients.

## Background

Meningioma is the most common primary intracranial tumor in adults and accounts for one-third (39.0%) of all primary intracranial tumors [1]. It is classified into three groups and 15 subgroups based on histopathological features by the current World Health Organization (WHO) classification published in 2021 [2]. Malignant meningiomas (MM) only account for 1–3% of all meningiomas and are classified as WHO grade III meningiomas [3]. MM displays a more aggressive biological behavior with accelerated tumor growth and higher tendency of brain invasion leading to poorer prognosis and higher recurrence rates compared to benign meningioma (BM) (WHO grade I) or atypical meningioma (AM) (WHO grade II). In reference to histopathology, MM are characterized by inherently high mitotic indices and a higher number of Ki-67 positive cells compared to WHO grade I-II meningiomas [4]. Extracranial metastases from meningioma of any grade are rare with an overall prevalence of 2% [5]. Several cases of contiguous occurrence of meningioma and cerebral metastasis have also been reported in the literature [6–11]. We present here an interesting case of a MM patient with metachronous metastases in the liver, lungs and bones and discuss proposed mechanisms for metastasis formation, histopathological differences and treatment modalities for metastatic MM.

## Case presentation

In 2018, a 47-year-old patient was admitted to our hospital with sudden-onset hemihypesthesia and paresthesia of the left body side. On magnetic resonance imaging (MRI) scan, an inhomogeneous, contrast agent enhanced, hyperintense tumor was visible on the right parafalcine vertex.

The patient had a medical history of a papillary meningioma WHO grade III on the parasagittal right side with occlusion of the superior sagittal sinus in 1998. Resection of the tumor mass and the superior sagittal sinus with interposition of the great saphenous vein was performed. Preoperatively, the patient had a severe visual impairment due to bilateral papilledema and advanced optic atrophy. Post-operatively, the patient suffered from complete vision loss and weakness of both legs, which was accompanied by a probably trauma-induced degeneration of the premotor cortex on imaging. Adjuvant radiation therapy of the resection cavity was administered with a daily dose of 1.8 Gy up to a total dose of 59.4 Gy in 1998. In 2016, the patient was shortly admitted to our hospital with a concussion. Computed tomography (CT) and MRI scan revealed a fracture of the parietal bone, but no relapse of the former diagnosed papillary meningioma could be detected. Until 2018, no signs of a local relapse were present, and the patient was mobile with the help of walking aids despite suffering from functional amaurosis. The patient had never been diagnosed with neurofibromatosis or other genetic disorders in the past.

**Fig. 1:**
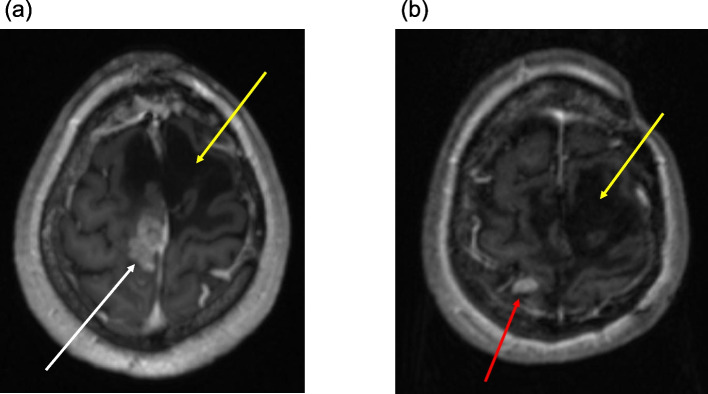
**a** MR T1 MPRAGE image with contrast agent acquired in 2018 at time of hospital admission with sudden-onset hemihypesthesia and paresthesia of the left body side. Contrast-enhancing parasagittal mass in the right parietal lobe with contact to the superior sagittal sinus (white arrow). **b** MR T1 MPRAGE image with contrast agent acquired in 2020 at follow-up. New contrast-enhancing mass in the right parietal lobe consistent with a relapse of the malignant meningioma (red arrow). Resection cavity after resection of a papillary meningioma WHO III in 1998 (yellow arrow)

**Fig. 2:**
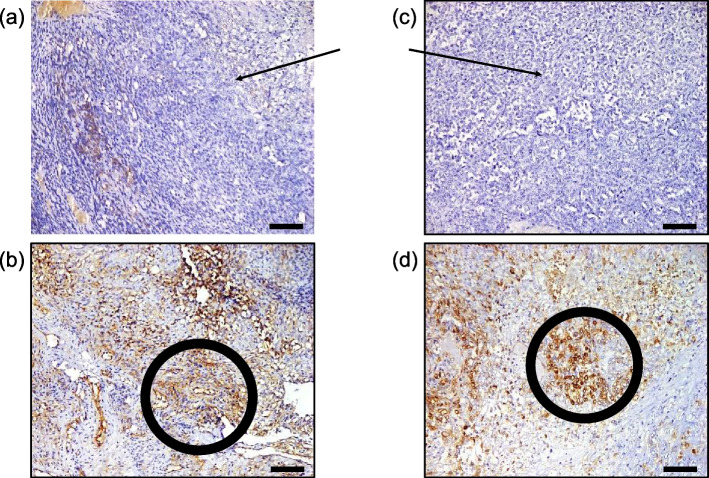
E-cadherin and beta-catenin staining of malignant meningioma specimens. **a** E-cadherin staining of the leptomeningeal resection with malignant meningioma cells (black arrow). Expression of E-cadherin is not detectable. **b** Beta-catenin staining of the leptomeningeal resection with malignant meningioma cells expressing beta-catenin (black circle). **c** E-cadherin staining of liver metastasis with malignant meningioma cells (black arrow). Expression of E-cadherin is not detectable. **d** Beta-catenin staining of liver metastasis with malignant meningioma cells expressing beta-catenin (black circle). Scale bar for all images: 200 µm

**Fig. 3:**
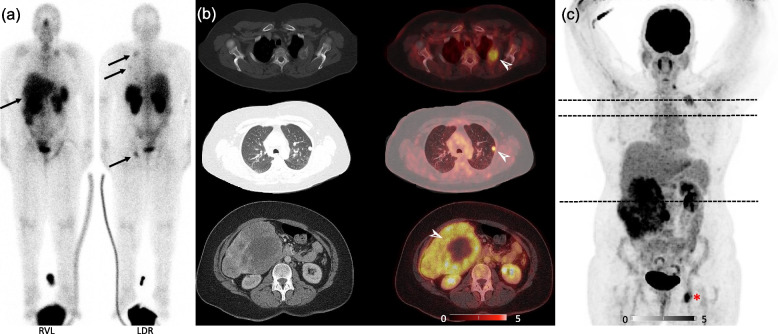
**a** Post-therapeutic whole-body scintigraphy after administration of 7.4 GBq Lu-177-DOTATOC showing suspicious radiotracer accumulation in lesions of liver, left lung and bone (black arrows). (b) Axial slices of CT and fused images with 18F-FDG PET of thorax and abdomen. Three tracer accumulations suspected of metastasis (osseous, pulmonary and hepatic) are highlighted (white arrowhead). **c** Maximum intensity projection (MIP) of 18F-FDG PET/CT. The dashed line marks the exemplary axial slices in B. Additional hypermetabolic lesions can be found for example in left iliac bone (red asterisk). *Abbreviations:* LDR = left-dorsal-right projection; RVL = right-ventral-left projection

When resubmitted to our hospital in 2018, the newly detected tumor was in close proximity to the dorsal edge of the previous resection cavity (Fig. 1a). After review of the case in the interdisciplinary neuro-oncological review board, an ultrasound-guided Simpson grade I resection of the tumor was performed under electrophysiological monitoring. Histopathological examination revealed an anaplastic meningioma WHO grade III (malignant meningioma) with epithelial membrane antigen (EMA) expression and a Ki-67 index of 15–20%. Histopathological results showed no epithelial cadherin (E-cadherin) expression but normal expression patterns of beta-catenin (Fig. 2a,  b). Due to prior cranial radiotherapy and Simpson grade I resection, radiation therapy was waived in the interdisciplinary neuro-oncological review board. In 2020, the patient had a relapse adjacent to the right postcentral gyrus (Fig. 1b). The relapsed tumor showed intense somatostatin receptor (SSTR) expression on ^68^ Ga-DOTA^0^-Phe^1^-Tyr^3^-octreotide (DOTATOC) positron emission tomography (PET) imaging. Since another resection was omitted due to adherence to two crucial bridging veins, the patient was presented to our clinic for a combined multimodal peptide receptor radionuclide therapy (PRRT) with sequential fractionated radiotherapy. After the first course of ^177^Lu-DOTATOC with an activity of 7.4 GBq, distribution scintigraphy routinely performed after PRRT revealed focal nuclide accumulations in the left lung, the liver and several bones as incidental findings (Fig. 3a). A ^18^F-fluorodeoxyglucose (FDG) PET/CT conducted to determine the extent of the lesions revealed a large hypermetabolic liver mass, hypermetabolic pulmonary metastases in the left upper lobe and in the right lower lobe, as well as multiple hypermetabolic osseous lesions (Fig. 3b, c). Histological sampling from the liver mass confirmed a metastatic lesion of the previously diagnosed MM, and atypical liver resection of segments IVb/V/VI was performed. E-cadherin and beta-catenin expression pattern were similar to the specimen from 2018 (Fig. 2c, d). No target mutations in anaplastic lymphoma kinase (ALK), B-Raf (BRAF), epidermal growth factor receptor (EGFR), fibroblast growth factor receptors (FGFR1/2/3), K-Ras (KRAS), mesenchymal-epithelial transition (MET), Neuregulin 1 (NRG1), rearranged during transfection (RET) and c-ros oncogene 1 (ROS1) genes were found in the molecular analysis of the specimen from the liver using Oncomine Comprehensive Assay v3 (OCA v3, Thermo Fisher Scientific Inc.) and FusionPlex Lung Panel (ArcherDX, Archer Inc.). Proficient mismatch repair (pMMR) loss and neurotrophic tyrosine receptor kinase (NTRK1/2/3) gene fusion were not detected. Programmed death-ligand 1 (PD-L1) tumor proportion score (TPS) was 4%, resulting in an immune cell (IC) score of 1. Due to increasing pain, radiotherapy of the left ischium, the left first and second ribs and lower thoracic/upper lumbar column was administered with a total dose of 30 Gy in 10 fractions each. In addition, the location of relapse was irradiated with a total dose of 39 Gy. After radiation therapy, the patient received two additional cycles of PRRT with 7.96 and 7.3 GBq ^177^Lu-DOTATOC. A month after application of the third cycle of PRRT, staging examination revealed a diffuse osseous and pulmonary metastatic progression with sintering of the eighth thoracic vertebra. The irradiated meningioma relapse showed no signs of progression. The patient received radiation therapy of parts of the thoracic column, the right acetabulum and the right forth rib with a total dose of 30 Gy in 10 fractions each. In addition, the cervical and lumbar column was irradiated with a total dose of 24 Gy in six fractions. PRRT could not be continued due to persistent pancytopenia under ongoing radiotherapy. Due to the palliative overall situation and deterioration of the general condition, the patient was transferred to palliative care unit after completion of the last radiotherapy. After further pain reduction and partial mobilization, the patient was discharged home and died a month later.

At discharge, the patient was satisfied with the achieved pain reduction but regretted the general course of the disease.

## Discussion and conclusions

Meningioma metastases occur in 2% of all patients with a higher incidence in MM [5]. A dissemination through the cerebrospinal fluid is rare, and hematogenous or lymphatic spread is even less common [12–18]. Iatrogenic contact metastases of meningioma after resection have also been described in the literature [19, 20]. The primary localization of meningioma is usually the central nervous system although ectopic extracranial manifestations of meningioma might also occur (< 2%), most frequently in the head and neck region [21]. It was suggested that ectopic meningiomas develop de novo from multipotent stem cells rather than resulting from a malignization of misplaced arachnoid cells although specific differences in the cytomorphology and immunohistochemical profile between ectopic and intracranial meningiomas could not be found [22]. In the majority of ectopic meningiomas, characteristic cytomorphological features include tightly cohesive clusters of spindled cells, whorls, intranuclear inclusions, nuclear grooves and psammomatous calcification. Unusual cytomorphological features including epithelioid cell predominance, abundance of inflammatory cells, small-cell changes, papillary structures and pseudoacinar growth could only be identified in a few cases. Compared to ectopic meningioma, metastatic meningioma appear to exhibit cellular atypia, increased mitotic rate, necrosis and apoptosis more frequently [23]. These cytomorphological features occur in AM and MM, which generally have a higher tendency to metastasize, but could not be found in the specimens of our patient [5].

The mechanisms of meningioma spreading are complex and not fully understood, however, loss or dysfunction of cell-to-cell adhesion molecules might be a crucial factor for invasion of tumor cells. Loss or malfunction of cell-to-cell adhesion molecules leads to a loss of intercellular connections between cells. Hence, contact inhibition is weakened which eventually drives uncontrolled growth of tumor cells and subsequently enables the invasion into surrounding tissues [24, 25]. Important cell adhesion molecules are E-cadherin and beta-catenin, which are part of the calcium-dependent E-cadherin/catenin complex that regulates cell adhesion and maintains cell stability. Its downregulation or malfunction results in a reduced adhesion between epithelial cells [24]. Although meningiomas are characterized by the expression of E-Cadherin together with Vimentin, more recent data showed a positive correlation between downregulation of E-cadherin and malignancy of meningioma [26–29]. Earlier reports, however, suggested no correlation [30, 31]. In regards to beta-catenin, the literature is incoherent. Beta-catenin appears to be upregulated with higher concentrations in the nucleus and cytosol, while expression levels in the membrane are downregulated [26, 28]. A complete loss of E-cadherin expression was found in the histopathological staining of our patient, which is concurring with previously published data (Fig. 2a, c) [26, 29]. Expression levels of beta-catenin in our specimens were normal (Fig. 2b, d).

Since most case reports were published before 2021, histopathological classification was based on outdated pathological characteristics, which raises the question if meningioma might have been falsely diagnosed in certain cases. For instance, solitary fibrous tumor (SFT), formerly known as solitary fibrous tumor/hemangiopericytoma (SFT/HPC), is associated with higher rates of recurrences and metastasis but has a similar presentation on imaging, resulting in a false diagnosis [32, 33]. At present, SFT can be distinguished from meningioma by the complete absence of EMA, nuclear expression of Signal Transducer and Activator of Transcription 6 (STAT6) and the detection of a NAB2-STAT6 fusion [34–36]. Due to the expression of EMA in the specimens of 2018 and 2020, the diagnosis of a SFT is very unlikely but cannot completely be excluded because STAT6 staining and gene sequencing for NAB2-STAT6 fusion were not performed. The development of a relapse or a radiation-induced secondary meningioma in 2018 is more likely. Radiation-induced meningioma tend to present as multiple high-grade tumors with an aggressive behavior and frequent tumor recurrence, fitting to the clinical course of our case report [37]. Other risk factors for meningiomas include female sex, diabetes mellitus, arterial hypertension and germline alterations in the Neurofibromin 2 (NF2) gene [38, 39]. Data are inconsistent with respect to smoking as a risk factor [39, 40]. A posttraumatic etiology of the meningioma in our case report is highly unlikely due to the minor severity of the trauma and the short time interval between trauma in 2016 and meningioma diagnosis in 2018. Published data suggest an average time interval between trauma and clinical manifestation of 12.95 years (range 4–45 years) [41].

European Association of Neuro-Oncology (EANO) guideline-compliant therapy for MM consists of a radical resection followed by adjuvant radiotherapy [42]. If the tumor is not resectable, primary radiotherapy is recommended. However, due to prior radiation therapy of the intracranial site in 1998, adjuvant radiotherapy for the patient was omitted in 2018 and was only applied at time of further relapse in 2021. In addition, no target-based therapies could be administered to the patient due to the lack of oncogenic mutations. Up to now, there is still no established systemic therapy for the effective treatment of recurrent meningioma of any grade. Options for pharmacotherapy include, but are not limited to, hydroxyurea, somatostatin analogues, vascular endothelial growth factor receptor (VEGF) antibodies, EGFR, platelet-derived growth factor receptor (PDGFR) and mammalian target of rapamycin (mTOR) inhibitors [43].

In our case report, an adequate pain relief was achieved at the sites of bone metastases by conventional fractionated radiotherapy with cumulative doses between 24–30 Gy, which are comparable to standard dose concepts for pain reduction in other entities.

Due to high expression levels of SSTR on the cell surface, meningiomas are often susceptible to SSTR-targeted PRRT, especially in combination with fractionated external beam radiation therapy [44]. Our patient received three cycles of ^177^Lu-DOTATOC with a cumulatively administered activity of 22.7 GBq. Whether PRRT therapy has delayed disease progression is difficult to prove in our case report. In a recent meta-analysis, disease progression risk in treatment-refractory meningiomas decreased by 13% per 1,000-MBq increase in the total applied activity but response rates were very poor for treatment-refractory MM [45]. In our case report, the patient developed new bone metastases 3 months after the first dose of PRRT but progression of the already existing metastases in the bones and the lungs could not be detected.

In our case report, the specimen of metastatic MM showed a histopathological downregulation of E-cadherin. E-cadherin downregulation seems to be associated with a higher probability of tumor invasion and distant metastasis formation in MM. Until now, the efficacy of systemic therapy, including PRRT, is very limited in malignant meningioma patients.

## Data Availability

The original contributions and data presented in the study are included in the manuscript. Further inquiries can be directed to the corresponding author.

## Abbreviations

ALK: Anaplastic lymphoma kinase
AM: Atypical meningioma
BM: Benign meningioma
BRAF: B-Raf
CT: Computed tomography
DOTATOC: DOTA^0^-Phe^1^-Tyr^3^-octreotide
EANO: European Association of Neuro-Oncology
EGFR: Epidermal growth factor receptor
EMA: Epithelial membrane antigen
FDG: Fluorodeoxyglucose
FGFR: Fibroblast growth factor receptor
IC: Immune cell
KRAS: K-Ras
MET: Mesenchymal-epithelial transition
MM: Malignant meningioma
MRI: Magnetic resonance imaging
mTOR: Mammalian target of rapamycin
NAB2: NGFIA-binding protein 2
NF2: Neurofibromin 2
NRG1: Neuregulin 1
NTRK: Neurotrophic tyrosine receptor kinase
PDGFR: Platelet-derived growth factor receptor
PD-L1: Programmed death-ligand 1
PET: Positron emission tomography
pMMR: Proficient mismatch repair
PRRT: Peptide receptor radionuclide therapy
RET: Rearranged during transfection
ROS1: C-ros oncogene 1
SFT: Solitary fibrous tumor
SFT/HPC: Solitary fibrous tumor/hemangiopericytoma
SSTR: Somatostatin receptor
STAT6: Signal Transducer and Activator of Transcription 6
TPS: Tumor proportion score
VEGF: Vascular endothelial growth factor receptor
WHO: World Health Organization

## References

[1] Ostrom QT, Cioffi G, Waite K, Kruchko C, Barnholtz-Sloan JS (2021). CBTRUS statistical report: primary brain and other central nervous system tumors diagnosed in the United States in 2014–2018. Neuro-oncology.

[2] Louis DN, Perry A, Wesseling P, Brat DJ, Cree IA, Figarella-Branger D (2021). The 2021 WHO classification of tumors of the central nervous system: a summary. Neuro Oncol.

[3] Maier H, Ofner D, Hittmair A, Kitz K, Budka H (1992). Classic, atypical, and anaplastic meningioma: three histopathological subtypes of clinical relevance. J Neurosurg.

[4] Daniela Maier A, Brøchner CB (2020). Mitotic and proliferative indices in WHO grade III Meningioma. Cancers (Basel).

[5] Dalle Ore CL, Magill ST, Yen AJ, Shahin MN, Lee DS, Lucas C-HG (2020). Meningioma metastases: incidence and proposed screening paradigm. J Neurosurg JNS.

[6] Bucciero A, del Basso de Caro M, Vizioli L, Carraturo S, Cerillo A, Tedeschi G (1992). Metastasis of breast carcinoma to intracranial meningioma. Case report and review of the literature. J Neurosurg Sci.

[7] Chatani M, Nakagawa I, Yamada S, Sugimoto T, Hironaka Y, Nakamura M (2014). Intracranial meningioma as initial clinical manifestation of occult lung carcinoma: case report. Neurol Med Chir.

[8] Miyagi N, Hara S, Terasaki M, Orito K, Yamashita S, Hirohata M (2007). A rare case of intracranial meningioma with intratumoral metastatic breast cancers. No shinkei geka Neurol Surg.

[9] Nakaya M, Ichimura S, Kurebayashi Y, Mochizuki Y, Fukaya R, Fukuchi M (2019). Contiguous metastasis of pulmonary adenocarcinoma to meningioma. J Neurol Surg Part A, Central Eur Neurosurg.

[10] Watanabe T, Fujisawa H, Hasegawa M, Arakawa Y, Yamashita J, Ueda F (2002). Metastasis of breast cancer to intracranial meningioma: case report. Am J Clin Oncol.

[11] Zon LI, Johns WD, Stomper PC, Kaplan WD, Connolly JL, Morris JH (1989). Breast carcinoma metastatic to a meningioma. Case report and review of the literature. Arch Int Med.

[12] Abboud M, Haddad G, Kattar M, Aburiziq I, Geara FB (2009). Extraneural metastases from cranial meningioma: a case report. Radiat Oncol.

[13] Lee GC, Choi SW, Kim SH, Kwon HJ (2009). Multiple extracranial metastases of atypical meningiomas. J Kor Neurosurg Soc.

[14] Cramer P, Thomale UW, Okuducu AF, Lemke AJ, Stockhammer F, Woiciechowsky C (2005). An atypical spinal meningioma with CSF metastasis: fatal progression despite aggressive treatment Case report. J Neurosurg Spine.

[15] Akimura T, Orita T, Hayashida O, Nishizaki T, Fudaba H (1992). Malignant meningioma metastasizing through the cerebrospinal pathway. Acta Neurol Scand.

[16] Wu JK, Kasdon DL, Whitmore EL (1985). Metastatic Meningioma to cervical vertebra: case report. Neurosurgery.

[17] Ludwin SK, Conley FK (1975). Malignant meningioma metastasizing through the cerebrospinal pathways. J Neurol Neurosurg Psychiatry.

[18] Shuangshoti S, Hongsaprabhas C, Netsky MG (1970). Metastasizing meningioma. Cancer.

[19] Messerer M, Nouri M, Saikali S, Brassier G, Hamlat A (2008). [Subcutaneous metastasis at the operative route of an atypical meningioma of the tentorium. Case report and literature review]. Neuro-Chirurgie.

[20] Ling M, Acharya J, Patel V (2020). Anaplastic meningioma seeding of the abdominal wall following calvarial bone flap preservation. Radiol Case Rep.

[21] Lang FF, Macdonald OK, Fuller GN, DeMonte F (2000). Primary extradural meningiomas: a report on nine cases and review of the CT-era literature. J Neurosurg.

[22] Rushing EJ, Bouffard J-P, McCall S, Olsen C, Mena H, Sandberg GD (2009). Primary extracranial meningiomas: an analysis of 146 cases. Head Neck Pathol.

[23] Ocque R, Khalbuss WE, Monaco SE, Michelow PM, Pantanowitz L (2014). Cytopathology of extracranial ectopic and metastatic meningiomas. Acta Cytol.

[24] Beavon IRG (2000). The E-cadherin–catenin complex in tumour metastasis: structure, function and regulation. Eur J Cancer.

[25] Arikkath J, Reichardt LF (2008). Cadherins and catenins at synapses: roles in synaptogenesis and synaptic plasticity. Trends Neurosci.

[26] Zhou K, Wang G, Wang Y, Jin H, Yang S, Liu C (2010). The potential involvement of E-cadherin and beta-catenins in meningioma. PLoS ONE.

[27] Wallesch M, Pachow D, Blücher C, Firsching R, Warnke J-P, Braunsdorf WEK (2017). Altered expression of E-Cadherin-related transcription factors indicates partial epithelial-mesenchymal transition in aggressive meningiomas. J Neurol Sci.

[28] Pećina-Šlaus N, Nikuševa Martić T, Deak AJ, Zeljko M, Hrašćan R, Tomas D (2010). Genetic and protein changes of E-cadherin in meningiomas. J Cancer Res Clin Oncol.

[29] Schwechheimer K, Zhou L, Birchmeier W (1998). E-Cadherin in human brain tumours: loss of immunoreactivity in malignant meningiomas. Virchows Archiv : Int J Pathol.

[30] Shimada S, Ishizawa K, Hirose T (2005). Expression of E-cadherin and catenins in meningioma: ubiquitous expression and its irrelevance to malignancy. Pathol Int.

[31] Figarella-Branger D, Pellissier JF, Bouillot P, Bianco N, Mayan M, Grisoli F (1994). Expression of neural cell-adhesion molecule isoforms and epithelial cadherin adhesion molecules in 47 human meningiomas: correlation with clinical and morphological data. Mod Pathol.

[32] Yamashita D, Suehiro S, Kohno S, Ohue S, Nakamura Y, Kouno D (2021). Intracranial anaplastic solitary fibrous tumor/hemangiopericytoma: immunohistochemical markers for definitive diagnosis. Neurosurg Rev.

[33] Ratneswaren T, Hogg FRA, Gallagher MJ, Ashkan K (2018). Surveillance for metastatic hemangiopericytoma-solitary fibrous tumors-systematic literature review on incidence, predictors and diagnosis of extra-cranial disease. J Neurooncol.

[34] Iwaki T, Fukui M, Takeshita I, Tsuneyoshi M, Tateishi J (1988). Hemangiopericytoma of the meninges: a clinicopathologic and immunohistochemical study. Clin Neuropathol.

[35] Schweizer L, Koelsche C, Sahm F, Piro RM, Capper D, Reuss DE (2013). Meningeal hemangiopericytoma and solitary fibrous tumors carry the NAB2-STAT6 fusion and can be diagnosed by nuclear expression of STAT6 protein. Acta Neuropathol.

[36] Chmielecki J, Crago AM, Rosenberg M, O'Connor R, Walker SR, Ambrogio L (2013). Whole-exome sequencing identifies a recurrent NAB2-STAT6 fusion in solitary fibrous tumors. Nat Genet.

[37] Al-Mefty O, Topsakal C, Pravdenkova S, Sawyer JR, Harrison MJ (2004). Radiation-induced meningiomas: clinical, pathological, cytokinetic, and cytogenetic characteristics. J Neurosurg.

[38] Preusser M, Brastianos PK, Mawrin C (2018). Advances in meningioma genetics: novel therapeutic opportunities. Nat Rev Neurol.

[39] Schneider B, Pülhorn H, Röhrig B, Rainov NG (2005). Predisposing conditions and risk factors for development of symptomatic meningioma in adults. Cancer Detect Prev.

[40] Flint-Richter P, Mandelzweig L, Oberman B, Sadetzki S (2011). Possible interaction between ionizing radiation, smoking, and gender in the causation of meningioma. Neuro Oncol.

[41] Caroli E, Salvati M, Rocchi G, Frati A, Cimatti M, Raco A (2003). Post-Traumatic Intracranial Meningiomas. Tumori Journal.

[42] Goldbrunner R, Stavrinou P, Jenkinson MD, Sahm F, Mawrin C, Weber DC (2021). EANO guideline on the diagnosis and management of meningiomas. Neuro Oncol.

[43] Brastianos PK, Galanis E, Butowski N, Chan JW, Dunn IF, Goldbrunner R (2019). Advances in multidisciplinary therapy for meningiomas. Neuro Oncol.

[44] Hartrampf PE, Hänscheid H, Kertels O, Schirbel A, Kreissl MC, Flentje M (2020). Long-term results of multimodal peptide receptor radionuclide therapy and fractionated external beam radiotherapy for treatment of advanced symptomatic meningioma. Clin Transl Radiat Oncol.

[45] Mirian C, Duun-Henriksen AK, Maier A, Pedersen MM, Jensen LR, Bashir A (2021). Somatostatin receptor-targeted radiopeptide therapy in treatment-refractory meningioma: individual patient data meta-analysis. J Nuclear Med.

